# The L-shaped association between weight-adjusted-waist index and all-cause mortality in individuals with psoriasis: results from NHANES database retrospective cohort study

**DOI:** 10.3389/fimmu.2025.1548788

**Published:** 2025-06-25

**Authors:** Jiawen Chen, Xueting Zeng, Niu Xiang, Renwei Luo, Zhixun Xiao, Rongying Chen, Beiqi Lin, Hui Ke, Ting Gong, Chao Ji

**Affiliations:** ^1^ Department of Dermatology, the First Affiliated Hospital of Fujian Medical University, Fuzhou, Fujian, China; ^2^ Institute of Dermatology, Fujian Medical University, Fuzhou, Fujian, China; ^3^ Fujian Dermatology and Venereology Research Institute, The First Affiliated Hospital, Fujian Medical University, Fuzhou, Fujian, China; ^4^ Key Laboratory of Skin Cancer of Fujian Higher Education Institutions, the Fujian Medical University, Fuzhou, Fujian, China; ^5^ Department of Dermatology, Shanghai Children's Medical Center, Shanghai Jiao Tong University School of Medicine, Shanghai, China

**Keywords:** weight-adjusted-waist indexes, all-cause mortality, psoriasis, obesity, L-shaped associations

## Abstract

**Background:**

While obesity is widely recognized as a robust risk factor contributing to both the onset and exacerbation of psoriasis—a chronic, immune-mediated skin disorder—the relationship between the Weight-Adjusted-Waist Index (WWI), an emerging metric for the nuanced evaluation of obesity, and mortality rates specifically within the psoriatic population is uncharted territory in current medical academic research. This study investigated the associations of WWI with all-cause mortality among American individuals with psoriasis.

**Methods:**

Data from the National Health and Nutrition Examination Survey (2003-2004, 2005-2006, 2009-2010, 2011-2012, and 2013-2014). Death outcomes were determined by linkage to National Death Index (NDI) records through December 31, 2019. Cox proportional hazards model and the two-piecewise Cox proportional hazards model were used to elucidate the nonlinear relationship between WWI and all-cause mortality in psoriasis patients.

**Results:**

A total of 577 participants were enrolled in the NHANES study, and 69 all-cause deaths occurred. After multivariable adjustment, higher WWI was significantly and nonlinearly associated with higher risk of all-cause mortality among participants with psoriasis. In addition, we found an L-shaped association between WWI and all-cause mortality, with WWI turning at 10.50cm/√kg for all-cause mortality. Among patients whose WWI was greater than the breakpoint, there was a 71% increase in the risk of death for each unit increase in WWI (HR 1.71; 95% CI 1.01, 2.88).

**Conclusions:**

Non-linear associations of WWI with all-cause mortality were observed in American patients with psoriasis, with a critical threshold of 10.50cm/√kg above which the risk of all-cause mortality increased significantly.

## 1 Introduction

Psoriasis is a chronic, non-communicable, inflammatory skin disease that causes pain, disfigurement, and disability. It affects over 60 million adults and children worldwide (1, 2). The condition significantly reduces the quality of life and is associated with comorbidities like obesity, metabolic syndrome, and cardiovascular disease, which impact physical, emotional, and social well-being (3). Consequently, individuals with psoriasis have a higher risk of mortality compared to those without the condition (4). Previous studies have shown that people with psoriasis have a 1.99-fold increased risk of death compared to those with non-psoriasis conditions (5). Therefore, identifying risk factors to decrease mortality rates is crucial for patients with psoriasis.

The weight-adjusted-waist index (WWI) is an anthropometric measure to assess central obesity. It is calculated by dividing waist circumference (WC) by the weight squared, taking into account both fat and muscle mass components. Importantly, WWI is applicable across different body mass index (BMI) categories, making it a valuable tool for assessing obesity (6). Numerous studies have shown WWI to be an independent and robust predictor of mortality, outperforming traditional measures such as BMI and WC (7, 8).

While the WWI is widely accepted as a reliable measure of central obesity, its association with mortality rates in psoriasis patients has remained conspicuously unexplored. Therefore, it becomes imperative to scrutinize the correlation between obesity-evaluated through the lens of WWI-and psoriasis, with the goal of enriching our collective understanding of deleterious effects of obesity on this patient cohort. By shedding light on this overlooked link, our research aims to raise awareness and stimulate scientific discourse about the profound impact obesity can have on the well-being of people with psoriasis.

## 2 Methods

### 2.1 Study design and population

Data for this study were collected from the National Health and Nutrition Examination Survey (NHANES), a cross-sectional, national, population-based survey conducted by the National Center for Health Statistics (NCHS) (9, 10). NHANES research protocols were approved by the NCHS Research Ethics Review Board (11). All participants in NHANES have provided informed consent, or for participants under the age of 16, consent was obtained from a parent and/or legal guardian. The datasets generated and analyzed for this study are publicly available on the NHANES website (https://www.cdc.gov/nchs/nhanes/index.htm).

**Figure 1:**
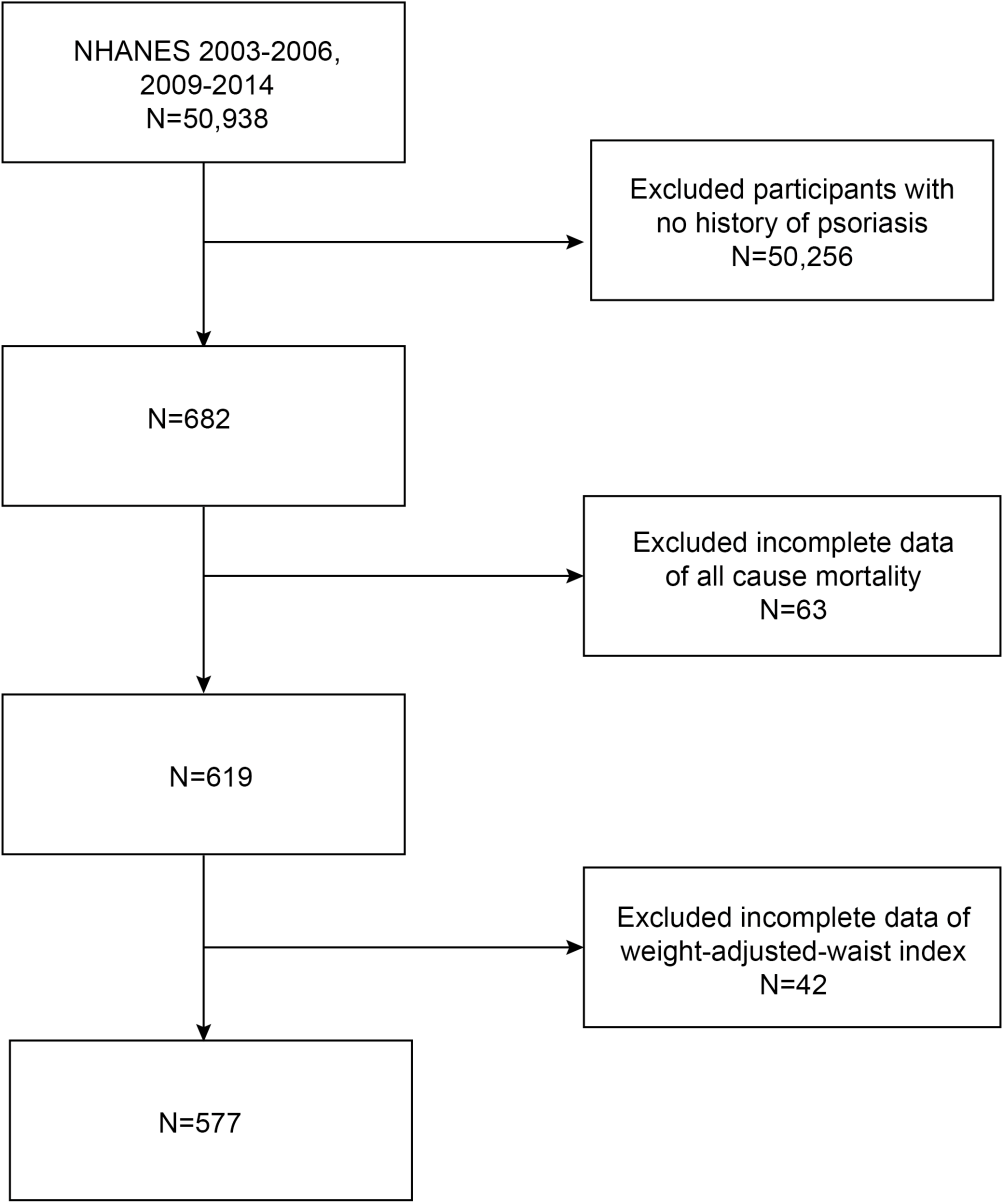
Flow chart of study participants.

Five NHANES cycles (2003-2004, 2005-2006, 2009-2010, 2011-2012, and 2013-2014) were downloaded to assess the association between WWI and all-cause mortality in patients with psoriasis. The exclusion criteria for the analysis of participants in our study were as follows: (1) individuals without a history of psoriasis, and (2) incomplete data regarding all-cause mortality and WWI. A total of 50,938 participants were initially enrolled. After excluding participants without a history of psoriasis (n=50,256), and those with missing data on all-cause mortality (n=63) and WWI (n=42), a final cohort of 577 eligible participants was included for analysis (**Figure 1**).

### 2.2 Definition of weight-adjusted-waist index

WWI, an anthropometric index calculated by dividing WC by the square of weight, serves as a specialized measure for the evaluation of central obesity. Recognized for its predictive prowess in prior mortality studies, WWI has demonstrated superiority over traditional metrics such as BMI and WC alone (6, 8). WWI was included as an exposure variable in our analysis.

### 2.3 Determination of mortality outcomes

Matching identifying information of patients to the National Death Index (NDI) was used for obtaining the mortality data on participants up to December 31, 2019.

### 2.4 Assessment of covariates

Covariates that may affect the association between WWI and all-cause mortality in individuals with psoriasis were included in our study as well, including gender (male/female), age (year), race (Mexican American/other Hispanic/non-Hispanic White/non-Hispanic Black/other races), education level (less than high school/high school or general educational development/above high school/others), family poverty income ratio (PIR), hypertension (yes/no), diabetes (yes/no), smoking (yes/no), arthritis (yes/no), cardiovascular disease (yes/no), total cholesterol (mg/dL) and triglycerides (mg/dL). All detailed measurement processes of these variables were publicly available on the NHANES website.

### 2.5 Statistical analysis

The statistical analysis performed in this study followed the guidelines of the Centers for Disease Control and Prevention (CDC). Continuous variables were summarized as means with standard errors (SE), while categorical variables were presented as proportions. Three Cox regression models were developed to estimate the association between WWI and hazard ratios (HRs) (95% CIs) for mortality in psoriatic patients: Model 1 (unadjusted); Model 2 was adjusted for gender, age, race, and survey cycle; Model 3 was adjusted for gender, age, race, survey cycle, education level, hypertension, total cholesterol, triglycerides, diabetes, smoking history, as well as histories of arthritis and cardiovascular disease (CVD). Subgroup analysis of the associations between WWI and all-cause mortality in psoriatic patients was performed with stratified factors including gender (male/female), age (<60/≥ 60 years), education level (high school or equivalent), marital status (married/others), smoking (yes/no), diabetes (yes/no), hypertension (yes/no), CVD (yes/no), arthritis (yes/no), total cholesterol (<200/≥ 200 mg/dL) and triglycerides (<150/≥ 150 mg/dL). These stratified factors were also treated as pre-specified potential effect modifiers. An interaction term was also added to test the heterogeneity of associations between the subgroups as well. All analyses were performed with R version 3.4.3 and Empower software 2.0. The level of statistical significance was set at P<0.05.

## 3 Results

### 3.1 Baseline characteristics of participants

A total of 577 participants were enrolled, of whom 46.79% were male and 53.21% were female, with a mean age of 48.84 ± 16.38 years. The ranges of WWI for quartiles 1–4 were 8.82–10.54, 10.54–11.10, 11.10–11.66, and 11.66-13.32, respectively. Among four WWI quartiles, differences with statistical significance were observed in age, race, education level, marital status, diabetes, hypertension, arthritis, CVD and triglycerides (all P<0.05). Subjects with increased WWI were female, older, had diabetes, hypertension, arthritis, CVD and higher triglycerides in our study (all P<0.05). Baseline characteristics of the study population according to WWI quartiles are shown in **Table 1**. To mitigate potential selection bias, a baseline comparison was additionally performed between participants excluded due to incomplete mortality and WWI data and the data that were included (see **Supplementary Table 1**).

**Table 1:** Baseline characteristics of study population according to weight-adjusted-waist index quartiles.

Weight-adjusted-waist index (cm/√kg)	Overall	Q1	Q2	Q3	Q4	*P*-value
N	577	144	144	144	145	
Age (years)	48.84 ± 16.38	38.09 ± 13.43	45.40 ± 13.06	52.45 ± 15.67	59.34 ± 15.14	<0.001
Gender (%)						0.006
Male	270 (46.79)	70 (48.61)	81 (56.25)	67 (46.53)	52 (35.86)	
Female	307 (53.21)	74 (51.39)	63 (43.75)	77 (53.47)	93 (64.14)	
Race (%)						0.001
Mexican American	48 (8.32)	6 (4.17)	7 (4.86)	12 (8.33)	23 (15.86)	
Other Hispanic	46 (7.97)	9 (6.25)	11 (7.64)	11 (7.64)	15 (10.34)	
Non-Hispanic White	351 (60.83)	92 (63.89)	89 (61.81)	86 (59.72)	84 (57.93)	
Non-Hispanic Black	72 (12.48)	24 (16.67)	12 (8.33)	22 (15.28)	14 (9.66)	
Other Races	66 (11.44%)	13 (9.03)	25 (17.36)	13 (9.03)	9 (6.21)	
Education level (%)						<0.001
Less than high school	112 (19.41)	21 (14.58)	14 (9.72)	35 (24.31)	42 (28.97)	
High school or GED	131 (22.98)	23 (16.43)	35 (24.82)	29 (20.14)	44 (30.34)	
Some college or AA degree	182 (31.93)	41 (29.29)	49 (34.75)	53 (36.81)	39 (26.90)	
College graduate or above	145 (25.44)	55 (39.29)	43 (30.50)	27 (18.75)	20 (13.79)	
Marital status (%)						<0.001
Married	300 (52.63)	68 (48.57)	85 (60.28)	80 (55.56)	67 (46.21)	
Widowed	35 (6.14)	3 (2.14)	3 (2.13)	11 (7.64)	18 (12.41)	
Divorced	72 (12.63)	10 (7.14)	22 (15.60)	13 (9.03)	27 (18.62)	
Separated	25 (4.39)	3 (2.14)	5 (3.55)	4 (2.78)	13 (8.97)	
Never married	89 (15.61)	44 (31.43)	12 (8.51)	20 (13.89)	13 (8.97)	
Others	49 (8.60)	12 (8.57)	14 (9.93)	16 (11.11)	7 (4.83)	
Diabetes (%)	72 (12.48)	4 (2.78)	10 (6.94)	18 (12.50)	40 (27.59)	<0.001
Hypertension (%)	243 (42.11)	27 (18.75)	49 (34.03)	65 (45.14)	102 (70.34)	<0.001
Smoking (%)	138	35 (49.30)	40 (47.62)	35 (49.30)	28 (49.30)	0.071
CVD (%)	82 (14.54)	6 (4.32)	12 (8.51)	25 (17.61)	39 (27.46)	<0.001
Arthritis (%)	234 (41.05)	32 (22.86)	51 (36.17)	70 (48.61)	81 (55.86)	<0.001
Cholesterol (mg/dL)	195.84 ± 41.39	189.91 ± 36.15	200.51 ± 43.35	195.93 ± 42.36	196.98 ± 43.01	0.364
Triglycerides (mg/dL)	159.04 ± 123.70	117.28 ± 69.84	176.37 ± 166.33	165.98 ± 123.52	176.40 ± 106.52	<0.001
All-cause mortality (%)	69 (11.96%)	6 (4.17)	9 (6.25)	19 (13.19)	35 (24.14)	<0.001

†Data are presented as mean ± SD or n (%). *GED* general educational development. *CVD* cardiovascular disease.

### 3.2 Relationships of WWI with mortality

During 5405.75 person-years of follow-up, 69 all-cause deaths occurred. We designed 3 Cox regression models to investigate the independent role of WWI in mortality. After multivariate adjustment including gender, age, race, survey cycle, education level, marital status, total cholesterol, total cholesterol, triglycerides, hypertension, and diabetes status. WWI was also positively associated with the odds of all-cause mortality with statistical significance as well and it was stable in our three models (**Table 2**). After full adjustment, subjects with one-unit higher WWI had a 9% increased risk of mortality (HR=1.09, 95% CI 1.09–1.10). When WWI categorized as quartiles, the multivariate-adjusted HRs and 95% CIs from lowest to highest WWI score (8.82–10.54, 10.54–11.10, 11.10–11.66 and 11.66-13.32) were 1.00 (reference), 0.34 (0.34, 0.34), 0.56 (0.56, 0.57), and 1.09 (1.09,1.10), respectively, for all-cause mortality (P for trend <0.0001).

**Table 2:** HRs (95% CIs) for mortality according to weight-adjusted-waist index among participants with psoriasis.

Weight-adjusted-waist index group	All-cause mortality		
	HR (95% CI)		
	Model 1^a^	Model 2^b^	Model 3^c^
Continuous	2.75 (2.75, 2.76)	1.61 (1.61, 1.61)	1.09 (1.09, 1.10)
Categories			
Q1	Reference	Reference	Reference
Q2	1.18 (1.17, 1.18)	0.82 (0.81, 0.82)	0.34 (0.34, 0.34)
Q3	2.56 (2.55, 2.57)	1.32 (1.31, 1.32)	0.56 (0.56, 0.57)
Q4	5.78 (5.76, 5.81)	2.04 (2.03, 2.05)	1.09 (1.09, 1.10)
*P* for trend	<0.0001	<0.0001	<0.0001

† ^a^Model 1: no covariates were adjusted. ^b^Model 2: adjusted for gender, age, race, and survey cycle. ^c^Model 3: adjusted for gender, age, race, survey cycle, education level, marital status, hypertension, total cholesterol, triglycerides, diabetes, smoking history, as well as histories of arthritis and CVD

**Figure 2:**
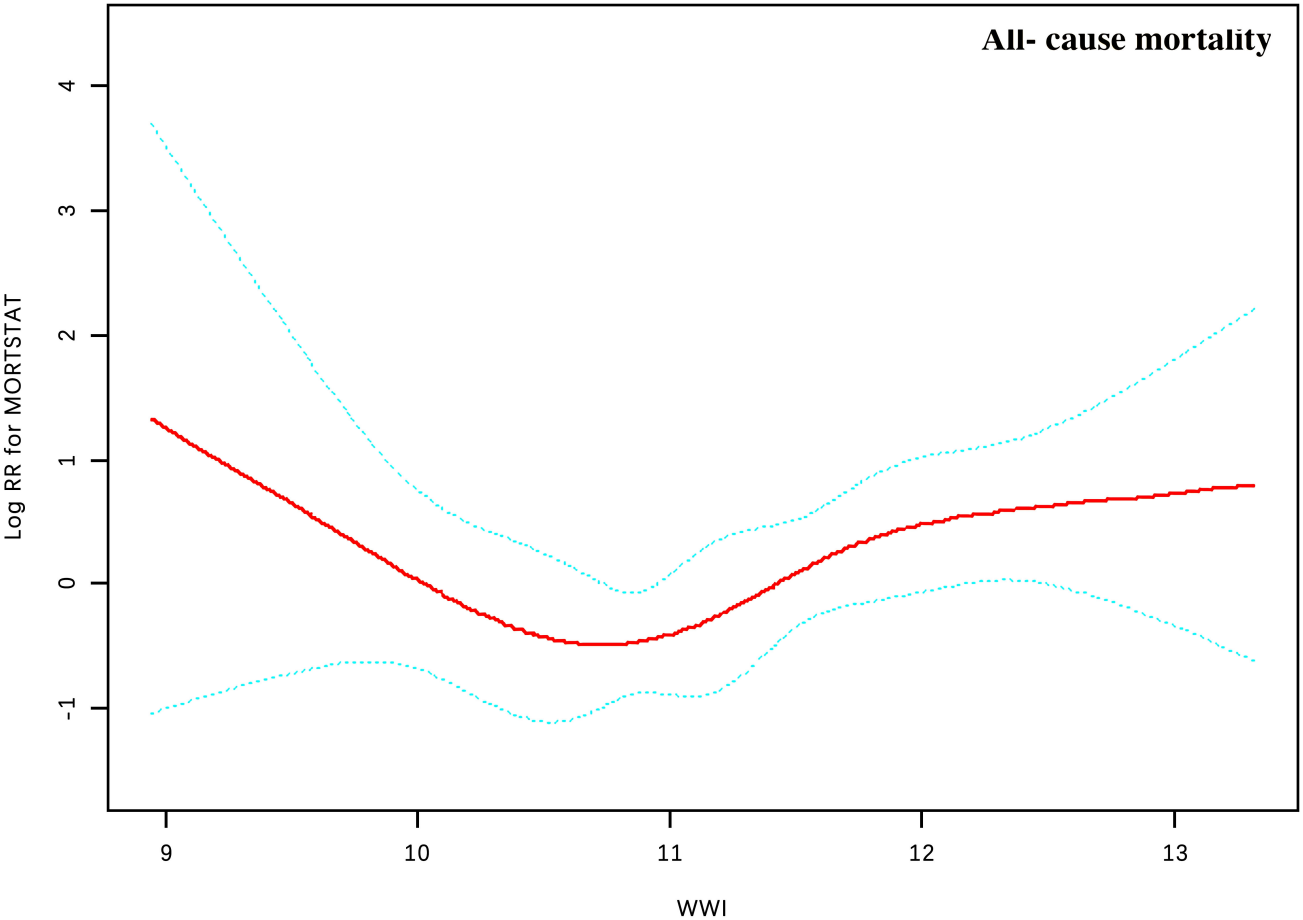
Association between WWI and all-cause mortality in patients with psoriasis. Adjusted for gender, age, race, survey cycle, education level, marital status, hypertension, total cholesterol, triglycerides, diabetes, smoking history, as well as histories of arthritis and CVD. The solid and dotted lines represent the estimated values and their corresponding 95% CIs, respectively.

By generalized additive models and smoothed curve fitting (penalized spline method), we discovered the L-shaped associations between WWI score concentrations and all-cause mortality (**Figure 2**). We then combined a Cox proportional hazards model with a two-piecewise Cox proportional hazards model to investigate the non-linear relationship between WWI score levels and all-cause in patients with psoriasis (P for log-likelihood ratio < 0.05) (**Table 3**). We further calculated the infection point (K) was 10.50cm/√kg. To the left of the infection point, no statistically significant association was found between WWI and mortality (HR 0.24; 95% CI 0.05, 1.05). On the right of the breakpoint, a 1-unit increase in the WWI score was associated with a 71% greater adjusted HR of all-cause with a positive relationship between WWI and mortality (HR 1.71; 95% CI 1.01, 2.88) was detected (**Table 3**).

**Table 3:** Threshold effect analysis of WWI concentrations on all-cause mortality in PSO patients.

Threshold Analysis Elements	Adjusted HR (95% CI), *P*-value
All-cause mortality	
Fitting by the standard linear mode	1.29 (0.82, 2.03) 0.2724
Fitting by the two-piecewise linear mode	
Inflection point	10.50cm/√kg
WWI score < 10.50cm/√kg	0.24 (0.05, 1.05) 0.0579
WWI score > 10.50cm/√kg	1.71 (1.01, 2.88) 0.0442
*P* for log-likelihood ratio	0.041

†Adjusted for gender, age, race, survey cycle, education level, marital status, hypertension, total cholesterol, triglycerides, diabetes, smoking history, as well as histories of arthritis and CVD.

### 3.3 Subgroup analysis

**Figure 3:**
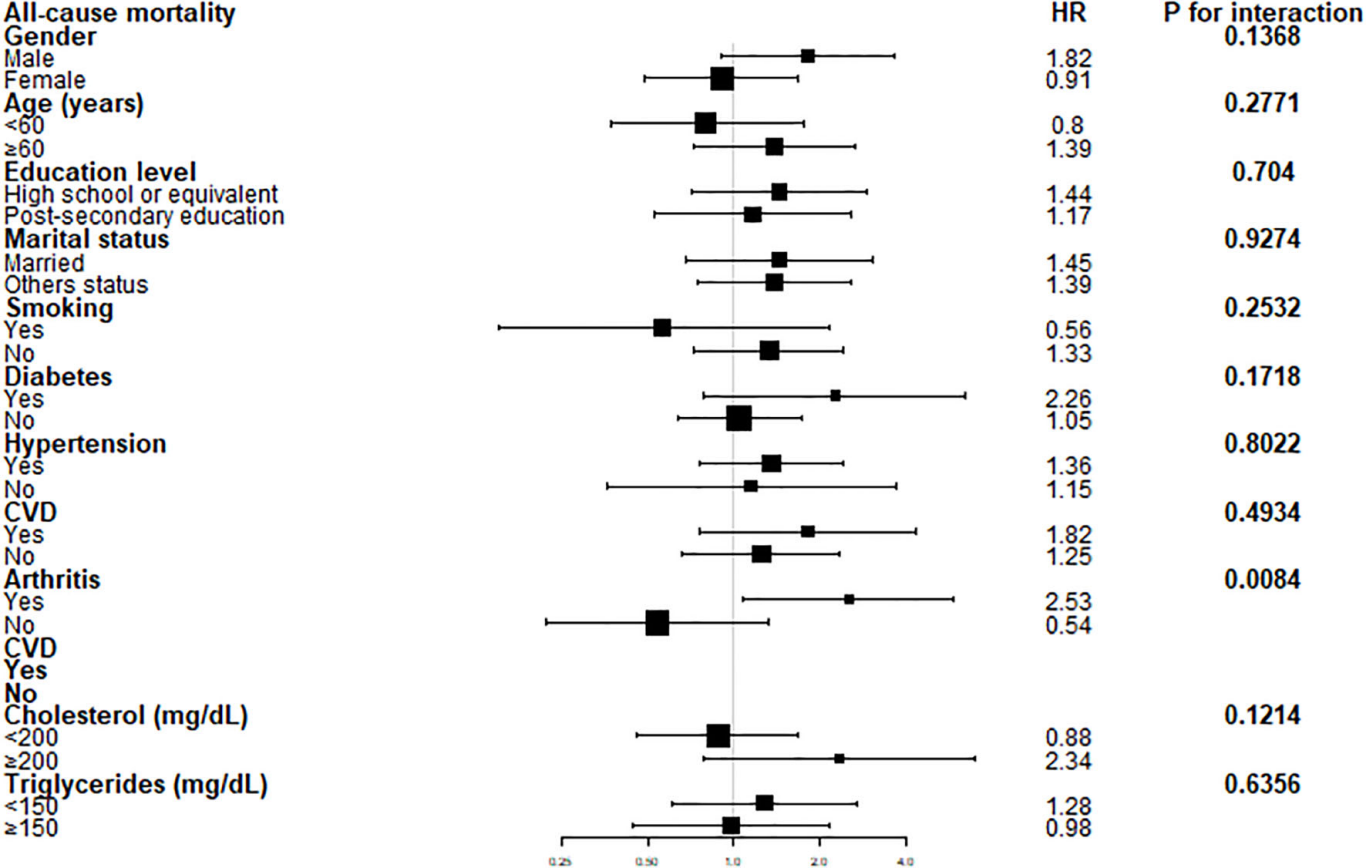
Forest plots of stratified analyses of WWI and all-cause mortality. Gender, age, education level, marital status, hypertension, total cholesterol, triglycerides, diabetes, smoking history, arthritis and CVD were all adjusted except for the variable itself. †Education level was classified as “High school or equivalent (less than high school, high school or GED)” and “Post-secondary education (Some college or AA degree, College graduate or above graduate degree).” Marital status was grouped as “Married” and “Others status (widowed, divorced, separated, never married, Others)”.

To evaluate whether the association between WWI and all-cause mortality in psoriatic patients was consistent in the overall population and for the potentially different population settings, we conducted subgroup analysis and interaction tests stratified gender, age, education level, marital status, hypertension, total cholesterol, triglycerides, diabetes, smoking history, arthritis and CVD. The advantage of a higher WWI score (>10.50cm/√kg) versus a lower WWI (<10.50cm/√kg) on survival of psoriatic patients was similar across a most of subgroups (**Figure 3**). Notably, our results showed that a stronger positive association between WWI and all-cause mortality was found patients with arthritis (P for interaction < 0.05).

## 4 Discussion

To our knowledge, the present retrospective study is the first analytical effort aimed at elucidating the association between WWI and all-cause mortality specifically in patients diagnosed with psoriasis. Our findings, characterized by the manifestation of unique 'L-shaped' trends, definitively underscore a statistically significant correlation between elevated WWI metrics and elevated rates of all-cause mortality within a circumscribed numerical range for this particular cohort of psoriatic patients. Such revelatory insights possess substantive implications, not merely affirming the instrumental role of WWI as an ancillary prognostic factor in assessing holistic mortality risk in psoriatic individuals but also foregrounding fertile avenues for future empirical inquiry within this underexplored domain of dermatological research.

As an immune-mediated chronic inflammatory disease, the inflammatory state in psoriasis has been exacerbated by obesity (12). Obesity, defined as a BMI of 30 or greater, is associated with shorter life expectancy due to a significantly increased risk of comorbidities and is known to be closely associated with psoriasis (4, 6, 13, 14). Previous research suggests that obesity worsens the risk of psoriasis and that individuals with psoriasis are more prone to secondary obesity. Consequently, this population may be at a higher risk of obesity-related complications, leading to increased mortality (15).

Intriguingly, several extant investigations have unearthed a phenomenon colloquially termed the 'obesity paradox’, where obesity, paradoxically, prognosticates a more favorable outcome compared to normative ranges of BMI (6, 8, 16, 17). WWI was proposed by Park et al. (8) initially to evaluate central obesity and discovered its positive association with mortality of cardiometabolic disease in the Korean population. Han et al. (7) found that WWI levels were associated with a higher risk of cardiovascular mortality and all-cause mortality independently in US adults. Ding et al. (16)also observed a non-linear positive relationship between WWI levels and cardiovascular and all-cause mortality.

Of particular note in our study was the identification of a non-linear relationship characterized by a discernible breakpoint at a WWI value of 10.50 cm/√kg. To the right of this breakpoint, a marked positive correlation with mortality was evident, whereas the left side showed an absence of any discernible correlation. This bifurcation suggests a threshold effect, indicating the critical role WWI may play in influencing mortality outcomes for patients with psoriasis. WWI thus emerges, by the evidence marshaled herein, as a potentially superior metric to both BMI and WC in the nuanced assessment of central obesity as a predictive factor for adverse prognoses.

The exact mechanisms underlying the positive association between higher WWI and mortality in psoriasis patients are not fully understood. However, the current understanding suggests that the detrimental effects observed with obesity, as indicated by elevated WWI, may be primarily attributed to disorders in lipid metabolism (18). Fatty tissue can produce pro-inflammatory factors that promote a state of chronic inflammation in the body. This chronic inflammation plays a significant role in the pathogenesis of psoriasis and is mediated by various cytokines, including Th1 and Th17 cells, which contribute to the sustained activation of immune responses and the development of the chronic inflammatory condition observed in psoriasis (4, 19).

There are some benefits to this study. First, we used a nationally representative sample of patients with psoriasis in the United States. The standardized data helps us to promote our results. Second, the number of mortality data in long-term follow-ups provided sufficient strength for the analysis in the present study. Furthermore, by adjusting for socioeconomic status, comorbidity, and other potential confounding factors, we could improve the effectiveness of the conclusion.

However, there are limitations to this study. Because it was an observational study, it could not determine cause and effect. Prospective research with larger sample sizes is necessary to clarify the causality. As in other observational studies, although we adjusted for covariates, we still could not avoid the effects of other confounding factors. Due to the limited data on deaths in patients with psoriasis in NHANES, this study only explored all-cause mortality and did not summarize other specific causes of mortality. In addition, NHANES is data from the United States, so we were only able to assess the association between WWI and mortality in the United States psoriasis patients. Because the study data came from only one country, data from other countries may be needed to further support this conclusion. Moreover, the inherent limitations of questionnaire-based surveys, such as recall bias and misreporting, may have affected some self-reported variables. Finally, the NHANES survey population may not fully represent all individuals with psoriasis, particularly those not engaged with the healthcare system, which could limit the generalizability of our findings. These issues warrant further investigation in our future research.

## 5 Conclusion

Upon rigorous multivariable adjustments, our study revealed a significant, non-linear association between elevated WWI and heightened risks of all-cause mortality in American psoriatic individuals, identified an 'L-shaped' trend with a critical inflection point at 10.50 cm/√kg—which could serve as a prospective intervention target for mortality risk mitigation—yet underscores the imperative for subsequent empirical validation of these provisional insights.

## Data Availability

The datasets generated and/or analyzed during the current study are available in the National Health and Nutrition Examination Survey repository, [https://www.cdc.gov/nchs/nhanes/index.htm].
