# Supplementary material for: The L-shaped association between weight-adjusted-waist index and all-cause mortality in individuals with psoriasis: results from NHANES database retrospective cohort study
Source: Front Immunol. 2025 Jun 25;16:1548788. doi: 10.3389/fimmu.2025.1548788 (PMC12238056; doi:10.3389/fimmu.2025.1548788)
Supplement: Supplementary file 1 [file Table1.docx]

| Supplementary Table 1 |  |  |  |
| --- | --- | --- | --- |
|  | **Inclusion group** | **Exclusion group** | ***P*-value** |
| **N** | 577 | 105 |  |
| **Age (years)** | 48.84±16.38 | 46.80±20.49 | 0.228 |
| **Gender (%)** |  |  | 0.343 |
| Male | 270 (46.8) | 55 (52.4) |  |
| Female | 307 (53.2) | 50 (47.6) |  |
| **Race (%)** |  |  | 0.759 |
| Mexican American | 48 (8.3) | 8 (7.6) |  |
| Other Hispanic | 46 (8.0) | 8 (7.6) |  |
| Non-Hispanic White | 351 (60.8) | 62 (59.0) |  |
| Non-Hispanic Black | 72 (12.5) | 18 (17.1) |  |
| Other Races | 60 (10.4) | 9 (8.6) |  |
| **Education level (%)** |  |  | 0.342 |
| Less than high school | 112 (19.6) | 25 (26.6) |  |
| High school or GED | 131 (23.0) | 16 (17.0) |  |
| Some college or AA degree | 182 (31.9) | 28 (29.8) |  |
| College graduate or above | 145 (25.4) | 25 (26.6) |  |
| **Marital status (%)** |  |  | 0.201 |
| Married | 300 (52.6) | 44 (46.8) |  |
| Widowed | 35 (6.1) | 12 (12.8) |  |
| Divorced | 72 (12.6) | 13 (13.8) |  |
| Separated | 25 (4.4) | 3 (3.2) |  |
| Never married | 89 (15.6) | 17 (18.1) |  |
| Others | 49 (8.6) | 5 (5.3) |  |
| **Diabetes (%)** | 72 (12.5) | 18 (17.1) | 0.253 |
| **Hypertension (%)** | 243 (42.1) | 6 (35.3) | 0.755 |
| **Smoking (%)** | 138 (42.2) | 19 (44.2) | 0.934 |
| **CVD (%)** | 82 (14.5) | 14 (14.9) | 1 |
| **Arthritis (%)** | 234 (40.6) | 34 (32.4) | 0.142 |
| **Abnormal cholesterol (mg/dL)** | 230.00 [214.00, 250.00] | 230.50 [210.25, 248.75] | 0.74 |
| **Triglycerides (mg/dL)** | 159.16±26.69 | 156.94±104.95 | 0.892 |

†Data are presented as mean ± SD, n (%), or median [IQR]. *Inclusion group* participants included in this study. *Exclusion group* participants excluded due to incomplete mortality and WWI data. *GED* general educational development. *CVD* cardiovascular disease. *Abnormal cholesterol* cholesterol level ≥150mg/dl.
